# Creating a more inclusive journal: the *Journal of the Medical Library Association*'s evolving process for selecting editorial board members

**DOI:** 10.5195/jmla.2022.1430

**Published:** 2022-01-01

**Authors:** Margaret Henderson, John W. Cyrus, Erin R. B. Eldermire, Jill T. Boruff, Katherine G. Akers, Beverly Murphy

**Affiliations:** 1 margaret.henderson@sdsu.edu, Margaret Henderson, AHIP, Former Editorial Board Member, San Diego State University, San Diego, CA; 2 cyrusiw@vcu.edu, John W. Cyrus, Editorial Board Member and Equity Workgroup Member, Virginia Commonwealth University, Richmond, VA; 3 erb29@cornell.edu, Erin R. B. Eldermire, Editorial Board Member and Equity Workgroup Member, Cornell University, Ithaca, NY; 4 iill.boruff@mcgill.ca, Jill T. Boruff, AHIP, Assistant Editor, McGill University, Montreal, Quebec, Canada; 5 kgakers@gmail.com, Katherine G. Akers, Former Editor-in-Chief and Equity Workgroup Member; 6 beverly.murphy@duke.edu, Beverly Murphy, AHIP, FMLA, Editorial Board Member and Equity Workgroup Member, Duke University, Durham, NC

**Keywords:** editorial board, equity, diversity

## Abstract

The *Journal of the Medical Library Association (JMLA)* selects new editorial board members every year. In the spring of 2021, *JMLA* used a new process for reviewing and selecting applicants for the limited number of open editorial board positions. This reevaluation of the selection process was spurred by a desire to create a more diverse and representative board. Changes to the procedures for selecting new editorial board members included having an open call for editorial board members, creating an application form, creating a selection committee to screen applicants, creating a form for the selection committee to extract data from applications, and creating a two-step process for screening and then selecting board members. As part of construction of this new process, areas for continued improvement were also identified, such as refining the application form to allow more specific answers to areas of interest to the selection committee. The newly created selection process for editorial board members constitutes a significant change in *JMLA* processes; however, more can be done to build on this work by further refining the selection process and ensuring that new members are selected in a transparent and streamlined manner.

The *Journal of the Medical Library Association (JMLA)* recently welcomed new editorial board members serving a 2021-2024 term using a new selection process focused on creating a more inclusive and diverse board. With transparency in mind, we wish to inform *JMLA's* readership about the impetus for changes to this process, how the process was changed, and how the process might be further improved in future years.

## WHY DID WE REEXAMINE THE EDITORIAL BOARD SELECTION PROCESS?

In response to national conversations in the summer of 2020 around systemic racism and health disparities, and in tandem with a larger diversity initiative within the Medical Library Association (MLA), *JMLA* began undertaking a series of actions toward becoming a more diverse, equitable, and inclusive journal [1]. The initial steps included issuing a statement of commitment to equity; a call for manuscripts addressing diversity, equity, and inclusion in health sciences librarianship; and the formation of an equity workgroup to coordinate this work

for the journal. As many readers know, this has not been a smooth process for *JMLA.* In particular, a group of Black authors who were invited to publish an editorial on anti-Blackness in librarianship [2] were treated inappropriately during the editorial process due to a lack of awareness and action on the part of *JMLA* editors and staff [3–5]. This led, ultimately, to the authors' decision to withdraw their manuscript, a public apology from the editor-in-chief [6], a couple of editorial board members quitting, and a change in *JMLA* staff. As a result, we are determined to learn from our mistakes and perform a deep interrogation of *JMLA's* processes and policies to prevent similar situations from occurring again and to make *JMLA* a better journal for all its stakeholders.

To inform our efforts to create more inclusive and equitable opportunities for authors, reviewers, and editorial board members, we conducted a demographic survey of all individuals who served as *JMLA* editorial board members or reviewers or had submitted a manuscript to *JMLA* between 2018 and 2020. We found that most respondents were white, heterosexual, women, and people without disabilities or impairments, similar to the demographic characteristics of the MLA membership [7]. While not altogether surprising, this finding suggests that *JMLA* lacked representation and contributions from individuals who do not meet the demographic “norm” of health sciences librarians and information specialists. Thus, having a more diverse editorial board is one way in which *JMLA* can be more inclusive of individuals with different racial and ethnic, sexual, gender, and disability identities and, in turn, benefit from their lived experiences and perspectives.

## HOW HAS THE PROCESS OF EDITORIAL BOARD MEMBER SELECTION EVOLVED?

Prior to 2016, the selection of *JMLA* editorial board members was conducted through standard MLA committee volunteer procedures. MLA members would fill out an application form and indicate their interests. Then, committee chairs and chair-designates looked through those applications along with the MLA staff member who supported that committee. Choices were then sent to the MLA president for approval, and letters were sent out. In addition to serving as an advisory board, *JMLA* editorial board members also served as the principal reviewers of manuscripts submitted to *JMLA.* In 2016, this process was reviewed and changed to reflect best practices in scientific publishing, in accordance with guidelines published by the Council of Science Editors, World Association of Medical Editors, and Committee on Publication Ethics (COPE), at the request of then-MLA President Teresa Knott [8]. As a result, the *JMLA* editor-in-chief is allowed to directly appoint editorial board members and to utilize a larger pool of external reviewers to comment and provide recommendations on submitted manuscripts. At that time, however, *JMLA* editorial board members still frequently performed peer review of submitted manuscripts. Therefore, ideal candidates for editorial board members were individuals who had their own record of peer-reviewed publications, experience serving as a peer reviewer, and subject expertise in needed areas.

In response to *JMLA's* editorial mistakes in December 2020, we revisited the process of selecting editorial board members again in the spring of 2021 with the primary goal of creating a more inclusive and diverse board. After discussions among *JMLA* editors and editorial board members, we collectively made several decisions.

First, we decided to remove peer review as a responsibility of editorial board members and to revise the role as follows: “The *JMLA* editorial board consists of individuals with diverse personal identities, professional roles, workplaces, and geographies who advise on journal processes and policies, act as journal ambassadors, and help keep the journal at the forefront of scholarly publishing. *JMLA* editorial board members attend virtual editorial board meetings and communicate through the journal's internal email listserv. They represent the journal in professional spaces and solicit manuscript submissions from authors. They can opt to join workgroups dedicated to specific initiatives (e.g., developing new policies or programs), to serve as liaisons to MLA Domain Hubs, and to act as mentors to editorial interns or new peer reviewers or authors. Editorial board members are expected to stay up-to-date with new developments in scholarly publishing and to actively participate in editorial board conversations and training opportunities'[9].

Second, we decided to explicitly consider individuals with a range of personal identities and professional roles, workplaces, and geographies. In recognition of the fact that only 31% of *JMLA* authors are MLA members [10], and in line with COPE's “Guidelines for the Board of Directors of Learned Society Journals” [11], we sought to recruit both MLA members and nonmembers as *JMLA* editorial board members to achieve broader representation of our stakeholders and ensure a degree of editorial independence from our parent association. In addition to health sciences librarians, we sought to include people in a variety of professional roles that support or are adjacent to health sciences librarianship (e.g., librarians and information specialists outside of health sciences, library staff, health care workers, researchers and educators in library and information science (LIS) and non-LIS disciplines, students, publishers/vendors). Recognizing that our authorship and readership is international [10], we also sought to recruit editorial board members from around the globe. Furthermore, to increase the demographic diversity of the *JMLA* editorial board, we sought to recruit individuals who identify as being from an underrepresented group in terms of racial or ethnic, gender, sexual, and disability identities.

Third, we decided to issue an open, wide-ranging call for applicants and task a committee with reviewing and selecting candidates to take a more inclusive and collaborative approach than that used in years past.

## WHAT IS THE NEW PROCESS OF SELECTING EDITORIAL BOARD MEMBERS?

A call for applicants for *JMLA* editorial board members for a 2021-2024 term was sent out in May 2021 via social media, email listservs, the *JMLA* website, and *MLAConnect.* Applicants were asked to complete an application form with their name, email address, institution name, position/role at that institution, country of residence, and answers to the following two sets of questions: (1) What experiences and/or qualities would you bring to the *JMLA* editorial board? How might you use these experiences and/or qualities to help improve *JMLA*'s processes, policies, and programs? and (2) What else do you want us to know? Full details of the application can be seen in Appendix 1.

We received ninety applications for between five and eight open positions on the editorial board. The selection committee, which consisted of outgoing editorial board members and members of the *JMLA* equity workgroup, quickly determined that a basic rubric for ranking applicants could not be applied to the pool, as our goal was to identify a deliberately diverse group of individuals based on several criteria. Therefore, the selection committee developed a screening form that allowed each committee member to extract specific information from the narrative portions of the applications into columns for various characteristics and to determine their top candidates for selection. The form was tested using several random applications, and adjustments were made where needed to increase clarity. A version of the final form used by the committee can be seen in Appendix 2. Selection committee members then started extracting data from the applications and screening applicants for final discussion and selection of new editorial board members.

Based on the quantity and quality of the pool of applicants, the selection committee realized that a key component of the selection process would revolve around how applicants answered the question “How might you use [your] experiences and/or qualities to help improve *JMLA's* processes, policies, and programs?” This became evident as some applicants wrote briefly about their experiences but did not expand on how those experiences would allow them to contribute to *JMLA.* Others, in contrast, gave thoughtful answers that showed their interest in the work of the editorial board and the goals of the journal. While the majority of applicants were removed from consideration based on their answer to this question, approximately 25% of the pool was recommended for consideration for a position on the editorial board. This remaining, smaller pool of applicants was then discussed by the selection committee via a Zoom meeting. Using data on the selection committee's answers to the question “Would you recommend this candidate for one of the 5–8 open *JMLA* editorial board positions?”, some new board members were unanimously recommended by all members of the selection committee, others were selected based on 75% agreement, and additional selected candidates had 50% agreement. Any applicant with less than 100% agreement was discussed by the committee in the context of the agreed-upon criteria and the goals of the selection process before coming to consensus.

Discussion of additional representation that would benefit the journal, including but not limited to stated experience in scholarship, publishing, or scholarly communication; self-identified membership in one or more underrepresented groups; primary discipline/field; area of librarianship (e.g., academic, public, hospital); career stage (e.g., student, new professional, experienced professional); and additional personal or professional experiences, factored heavily into the consideration of nominated candidates. The nominated candidates were then passed on to the editor-in-chief, who extended formal invitations. Happily, all nominated candidates enthusiastically accepted the invitation to serve as *JMLA* editorial board members.

## HOW MIGHT THIS PROCESS BE FURTHER IMPROVED?

Although we dramatically changed the process for selecting new *JMLA* editorial board members this year, considerable work remains to be done. The final action of the selection committee was to pass on recommendations for improvements to this process with the goal of making it more efficient, transparent, and inclusive.

1. The application form should be revised to more directly obtain demographic information from applicants. For instance, in addition to fields for institution name, position/role at that institution, and country of residence, the application could include fields for MLA membership status as well as race or ethnicity, gender, sexual, and disability identities. This would streamline the selection of candidates who would increase the diversity of the editorial board in terms of professional and personal characteristics.
2. The free-text question “What experiences and/or qualities would you bring to the *JMLA* editorial board? How might you use these experiences and/or qualities to help improve *JMLA*'s processes, policies, and programs?” on the application should be split into two separate questions. Distinguishing between these two questions could reinforce the importance of applicants addressing both questions and allow applicants the opportunity and space to do so.

In addition to these practical recommendations, we hope that *JMLA* will continue to revisit and hone the process of selecting new editorial board members and will continue to enable a selection committee with diverse representation to undertake this work. Ultimately, we know that only by continuing to reflect on this process and remaining open and attentive to improving our work can we achieve a *JMLA* editorial board that fully represents our audience.

## SUPPLEMENTAL FILES

**Appendix 1.** Questions on application form

**Appendix 2.** *JMLA* board selection criteria form fields
