# Supplementary material for: Creating a more inclusive journal: the Journal of the Medical Library Association's evolving process for selecting editorial board members
Source: J Med Libr Assoc. 2022 Jan 1;110(1):1–4. doi: 10.5195/jmla.2022.1430 (PMC8830382; doi:10.5195/jmla.2022.1430)
Supplement: Supplementary file 1 — Appendix 1. Questions on application form [file jmla-110-1-1-s01.docx]

Appendix 1

Questions on application form

1. Name
2. Email address
3. Institution
4. Position/role at your institution
5. Country of Residence
6. What experiences and/or qualities would you bring to the *JMLA* editorial board? How might you use these experiences and/or qualities to help improve *JMLA*'s processes, policies, and programs? [1,500 characters maximum]
7. What else do you want us to know? [1,500 characters maximum]
