# Supplementary material for: Creating a more inclusive journal: the Journal of the Medical Library Association's evolving process for selecting editorial board members
Source: J Med Libr Assoc. 2022 Jan 1;110(1):1–4. doi: 10.5195/jmla.2022.1430 (PMC8830382; doi:10.5195/jmla.2022.1430)
Supplement: Supplementary file 2 — Appendix 2. JMLA board selection criteria form fields [file jmla-110-1-1-s02.docx]

Appendix 2

*JMLA* board selection criteria form fields:

Q1. Applicant First Name *

Q2. Applicant Last Name *

Q3. Does the applicant answer the question "How might you use these experiences and/or qualities to help improve *JMLA*'s processes, policies, and programs?" or in some way indicate why they are interested in this position. If you've answered NO, then you do not need to answer the other questions for that person *

Mark only one.

Yes

No

Q4. Does the candidate self-identify as an underrepresented group or diverse personal identity?

Check all that apply.

BIPOC

LGBTQIA+

person with a disability

Other:

Q5. Role or job

Check all that apply.

Librarian

Other information professional

Health care professional (nurse, doctor, therapist, etc.)

Educator

Publisher

Student

Other:

Q6. Workplace—for librarians

Check all that apply.

Medical library

Academic library

Special library

Public library

Non-library

Other:

Q7. MLA member?

Mark only one

Yes

No

Q8. Location

Mark only one

Africa

Asia

Australia

Canada

Central/ South America

Europe

United States

Q9. Knowledge or Experience

Check all that apply.

Journal editorial board

Journal editor

Author

Reviewer

Publisher

Writer

Policies

Research

Scholarly communication

Expressed interest in publishing

Expressed interest in increasing diversity or social justice

Other:

Q10a. Would you recommend this candidate for one of the 5–8 open *JMLA* editorial board positions? (NOTE: please don't choose more than 15 Yes candidates) *

Mark only one.

Yes

Maybe

No

Other:

Q10b. Optional. If Yes, what makes you think this person would be a good editorial board member. Or any other comment you might have about this applicant that would help our decision-making.
